# In silico studies reveal structural deviations of mutant profilin-1 and interaction with riluzole and edaravone in amyotrophic lateral sclerosis

**DOI:** 10.1038/s41598-021-86211-4

**Published:** 2021-03-25

**Authors:** Ahmad Shahir Sadr, Changiz Eslahchi, Alireza Ghassempour, Mahmoud Kiaei

**Affiliations:** 1 Department of Computer and Data Sciences, Faculty of Mathematical Sciences, Shahid Beheshti University, Tehran, Iran; 2 School of Biological Sciences, Institute for Research in Fundamental Sciences (IPM), 193955746, Tehran, Iran; 3 Department of Phytochemistry, Medicinal Plants and Drugs Research Institute, Shahid Beheshti University, Tehran, Iran; 4 Department of Pharmacology and Toxicology, College of Medicine, University of Arkansas for Medical Sciences, Little Rock, AR 72205 USA; 5 Department of Neurology, College of Medicine, University of Arkansas for Medical Sciences, Little Rock, AR 72205 USA; 6 Department of Geriatrics, College of Medicine, University of Arkansas for Medical Sciences, Little Rock, AR 72205 USA; 7 RockGen Therapeutics, LLC., c/o Bioventures, LLC, 4301 W. Markham St., #831, Little Rock, AR 72205 USA

**Keywords:** Computational biology and bioinformatics, Neuroscience, Biomarkers

## Abstract

This study aimed to investigate four of the eight PFN-1 mutations that are located near the actin-binding domain and determine the structural changes due to each mutant and unravel how these mutations alter protein structural behavior. Swapaa’s command in UCSF chimera for generating mutations, FTMAP were employed and the data was analyzed by RMSD, RMSF graphs, Rg, hydrogen bonding analysis, and RRdisMaps utilizing Autodock4 and GROMACS. The functional changes and virtual screening, structural dynamics, and chemical bonding behavior changes, molecular docking simulation with two current FDA-approved drugs for ALS were investigated. The highest reduction and increase in Rg were found to exist in the G117V and M113T mutants, respectively. The RMSF data consistently shows changes nearby to this site. The *in silico* data described indicate that each of the mutations is capable of altering the structure of PFN-1 *in vivo*. The potential effect of riluzole and edaravone two FDA approved drugs for ALS, impacting the structural deviations and stabilization of the mutant PFN-1 is evaluated using *in silico* tools. Overall, the analysis of data collected reveals structural changes of mutant PFN-1 protein that may explain the neurotoxicity and the reason(s) for possible loss and gain of function of PFN-1 in the neurotoxic model of ALS.

## Introduction

Amyotrophic lateral sclerosis (ALS) is an inexorable neurodegenerative disease and is the subject of multiple worldwide intense investigation to determine the mechanisms for motor neuron death and treatment development to cure or slow the progression of the disease^1^. ALS (also known as Lou Gehrig’s disease) can progress quickly with a life expectancy of only 3–5 years after diagnosis and is considered as one of the devastating diseases. Due to the selective loss of upper and lower motor neurons, ALS Patients become progressively weak (mainly muscle weakness), and ultimately will cause death. ALS is largely sporadic (~ 90–95%) and only a small fraction of patients have the familial forms (fALS) (5–10%), with an estimated overall incidence of 2–4 per 100,000, with the ratio of 1:1.4 women vs men^2^.

Studies on the DNA samples from fALS patients led to the discovery of mutations in numerous genes associated with the disease and involved in the mechanism of neurodegeneration^3^. In this study, we bring focus to a recent and important mutation in such a molecule with the potential to unravel and shed light on the mechanism in axonal pathology, cytoskeletal importance in motor neurons in ALS. This molecule is the profilin-1 gene (*PFN-1*). Mutations in the PFN-1 have been identified as one of the genetic causes for fALS^4,5^. The protein expressed from the *PFN-1* gene is named PFN-1 and it is a multifunctional protein. PFN-1 is a critical protein in the conversion of monomeric (G)-actin to filamentous (F)-actin^6^. The approach used in this study has the power and capability to generate critical data towards unraveling the structural deviations and shed necessary light on the mechanisms underlying mutant PFN-1 neurotoxicity in ALS. The computational techniques on this approach are based on the report by Potapov *et al*.^7^. These in silico tools enabled us to investigate the structure of PFN-1 in the wild-type and mutant forms, rapidly and cost-effectively. In contrast to conventional approaches, the usable data generated and available from clinical trials at considerable expense and time and the pre-clinical studies that require the use of a large number of animals are significantly burdensome that in silico studies have the edge^8^. Albeit none of these approaches can provide all the answers needed to swiftly produce FDA approved medicine to treat a disease such as ALS. We posit that both approaches are needed and should be used with accuracy and precision.

Systems biology utilizes modular bioinformatics platforms to enable studies of molecular interactions at specific experimental setups and procedures that identify subcellular machinery responsible for specific functions in cells, tissues, and organ systems resulting in physiological behaviors^9,10^. This bioinformatics-system integrated interface considers modularity in systems biology and uses the biophysics-based and molecular mechanics tools with the choice on the selected functionally which becomes a vital component in the building and understanding of a circuit or a machine^11^.

In this study, the perturbations caused by these four mutations (C70G, M113T, E116G, and G117V) on the actin-binding area of the PFN-1 structure were the subject of investigation and we examined how they may impact its function that leads to a toxic “gain/loss of function”. There is a sense of urgency to study for better understanding and facilitating the possible treatment for this disease. We report our investigations on mutant PFN-1 using computational analytical tools under specific and pre-determined conditions, tested, and carried data analysis using computer-aided tools, data mining approaches, and systems biology. It is of great interest and importance to investigate the effect of the other 4 mutations (A19T, T108M, R135W, Q138L) on the PLP domain of PFN1, which will be pursued in the future.

## Methods and materials

**Figure 1:**
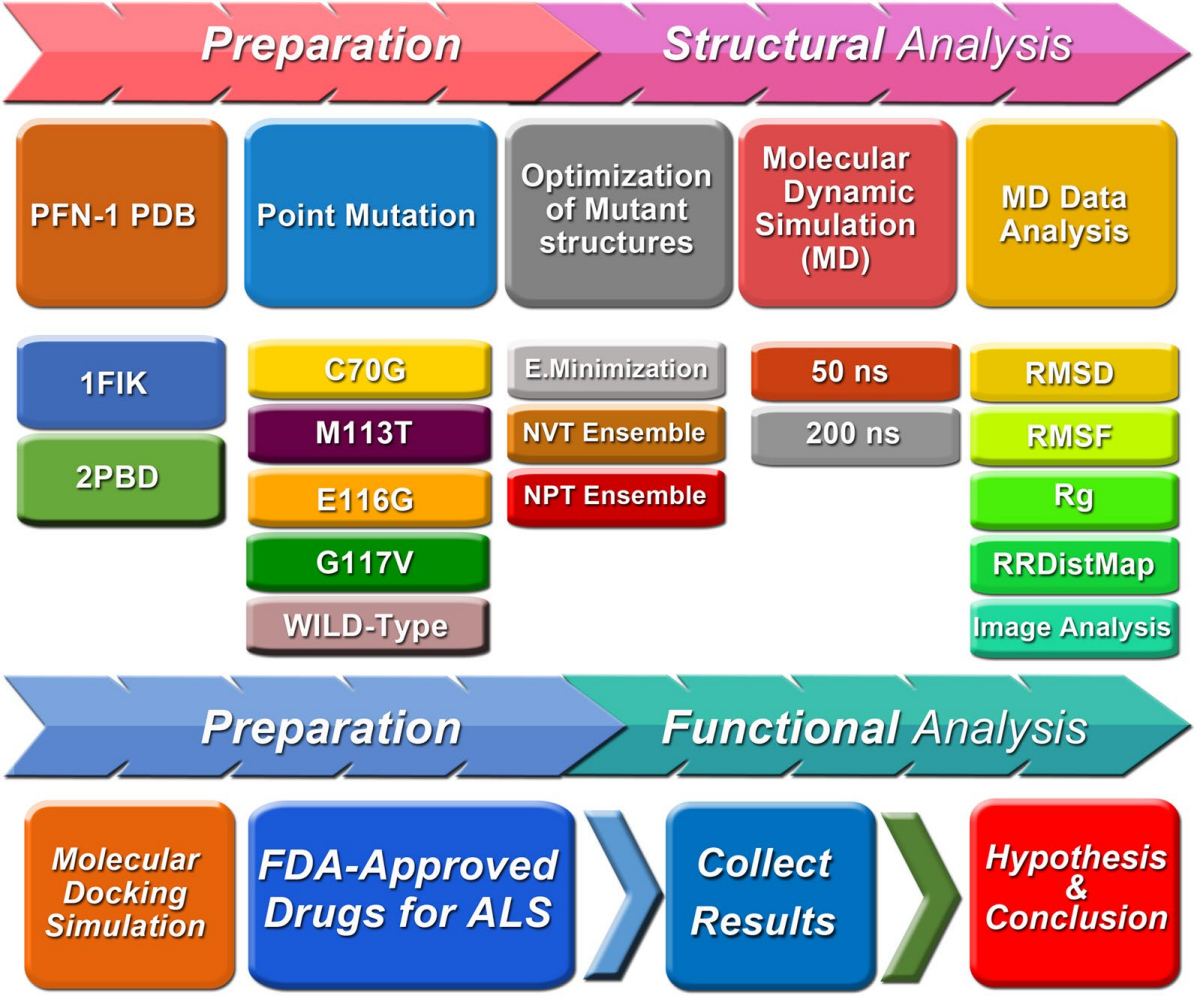
A diagrammatic summary of the preparation, structural and functional analysis of PFN-1. The PDB file of PFN-1 obtained from the protein data-bank, 4 point mutations under the investigation added one at a time, optimized, performed molecular dynamic simulation, and data analysis carried out as outlined in the cartoon format. The lower part of the diagram demonstrates the outline of our work with respect to the effect of the two FDA approved drugs for ALS.

A comprehensive in silico analysis was employed for this study by utilizing a variety of bioinformatics tools including software and databases. The structure of wild-type and mutant PFN-1 was examined, and the changes were explored at depth to shed light on potential reasons for the mutant PFN-1’s toxicities in ALS (Fig. 1).

### Wild-type PFN-1

Protein database searches in RCSB.org were performed using compiled crystal structure data for PFN-1. Amongst the 168,599 biological molecules’ crystal structures and macromolecules registered in the protein data-bank to date, 15 of them belong to PFN-1 (Supplementary Table S1).

These 15 PFN-1 crystal structures that are registered in the protein data-bank (Rcsb.org) includes two structures with mutations (4X1M, 4X25), the other nine structures are linked to other proteins such as polyproline chain containing proteins (e.g. VASP) and actin (2PAV, 2PBD, 3CHW, 1AWI, 1CF0, 1CJF, 6NAS, 6NBW, 6NBE) binding proteins. The other four structures of PFN-1 unbound to any other protein or macromolecules, their amino acid chains crystal structure data are fully resolved (1FIK, 1FIL, 1PFL and 4X1L). We have considered studying two of the crystal structures; 1FIK code from the unbounded set, contains one molecule in the asymmetric unit with diffraction of at least 2.0 Å and 2PBD code from the bounded set as a profilin-actin complex structure (Fig. 1).

### PFN-1 protein data base identification (PDBID): 1FIK

The crystal structure of PFN-1 coded 1FIK that is unique in the protein chain and has a phosphate ion and several water molecules bound to it and analyzed in this study. Our initial observation and analysis of the structure using the University of California San Francisco (UCSF) Chimera program^12^, gave us the notion of removing excess molecules of phosphate and water. This approach enabled us to create PFN-1 protein data that can be easily entered into the molecular dynamics (MD) simulation environment platform. The process of crystallography typically exerts stress on proteins due to reduction in humidity, and temperature change may affect and cause bias to the actual structure of the protein^13^. To obtain a more realistic structure close to its de novo state, we used a molecular dynamics simulation method and subsequently investigated the structural changes in PFN-1 by subjecting PFN-1 to 200 ns (ns) in MD simulations.

### Molecular dynamics simulation

All MD simulations calculations were performed using GROMACS software (Ver.2018)^14^ and Ubuntu operating system (OS) Ver.16^15^. The polarizable simulations were carried out in a 3D cubic box as a unit cell with a simple point charge (SPC) water model. The protonation state of ionizable amino acids with pKa of any given amino acid is assumed at pH 7. The positive and negative ions were added to the solvent to neutralize the surface charge of the molecule and to establish normal conditions; for example, we added 2 negative ions of chlorine (Cl) to neutralize the total of 9202 water molecules in the formed space as a solvent. The Optimized Potentials for Liquid Simulations for all atoms force field model also has been developed and used in MD simulation series. The molecules were used as solvents in the aquatic environment with the SPC model. For the residue interactions, the thickness of this solvent layer is assumed to be fixed with a threshold of at least 10 Å.

We also ensured that no steric clashes or inappropriate geometry. in a solvated system of PFN-complexes exist. On the other hand, we were mindful of the fact that performing simulations without the energy minimization step, may result in higher energy creation and disruption of structure. This phenomenon is due to the addition of hydrogen and breakage of the hydrogen bond network in the water. Therefore, to eliminate these unwanted forces, the structure is relaxed through a process called energy minimization (EM). To avoid (protein) position deviation during MD simulations, it was first performed during a 50,000 spatial limiting step as NVT and NPT ensembles equilibration where N is the number of molecules, V is the volume of the system, T is the temperature and P is the pressure. These were used to stabilize the temperature, the pressure, and the density of the system, respectively. Equilibration of pressure was conducted under an NPT ensemble, wherein the number of particles, pressure, and temperature were all kept constant^16^. Finally, softwares such as Qt Grace (is a tool to make two-dimensional plots of numerical data, generate graphs and analyze the results)^17^ and UCSF Chimera were used to further visualize and analyze the data. All the simulations were repeated three times for each set of parameters and in many independent runs.

### Modeling and optimization, structures

**Figure 2:**
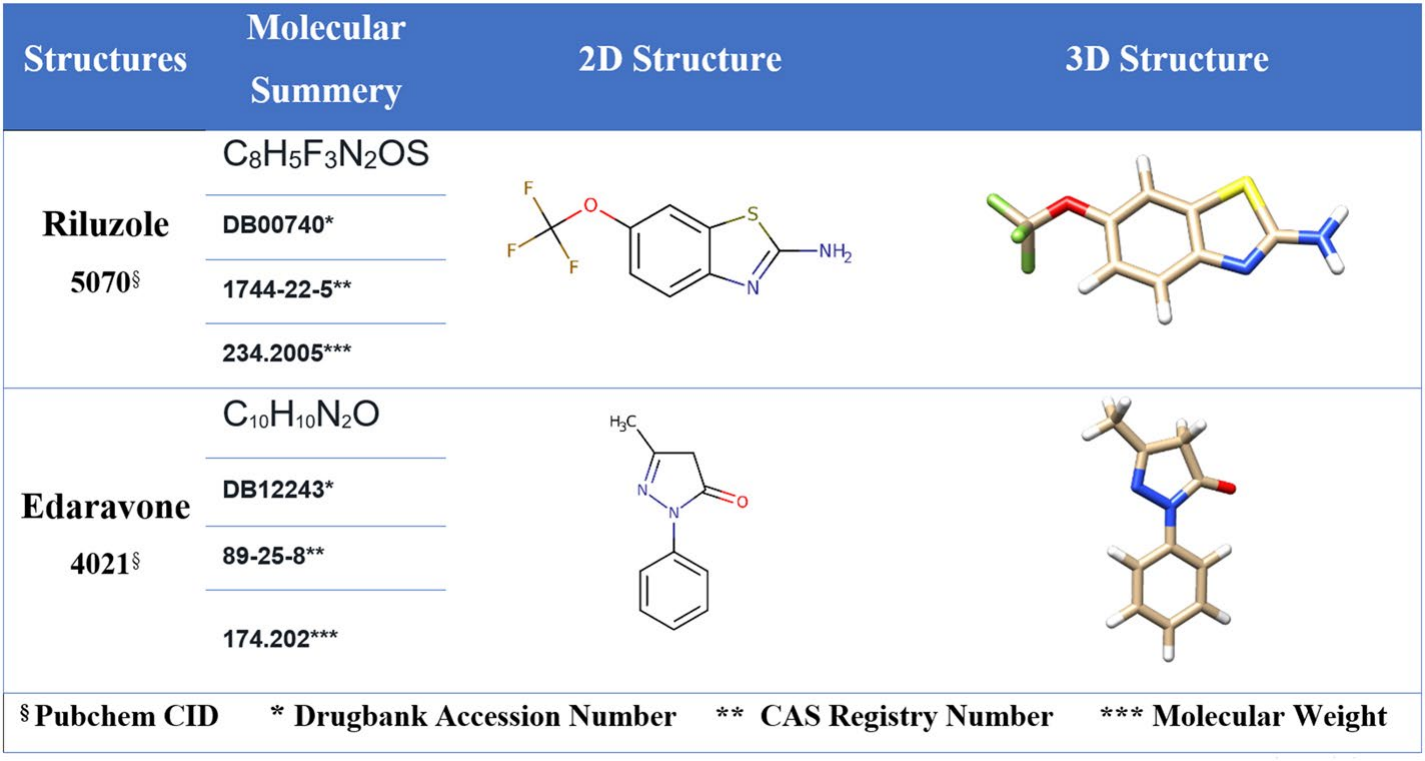
Two-dimensional and three-dimensional view of ALS FDA-approved drugs (riluzole and edaravone) before and after B3LYP protocol optimization of spatial energy of the Gaussian program. The structure of the (**A**) Riluzole and (**B**) Edaravone were obtained from the Drug bank (www.drugbank.ca).

The molecular structures required in this study were obtained from the PubChem structural database^18^ with their unique chemical identification number (CID) and CAS number as numerical identifier assigned by chemical abstract service (CAS) as listed in Fig. 2. Then, we attempted to optimize the structural energy to obtain the proper spatial geometry structures. Further, the GaussView program (ver. 05)^19^ provided the necessary outputs for the Gaussian quantum steering calculations (ver. 03) package, while making initial changes. In this program, we obtained the optimal structure by using the DFT/B3LYP protocol by command line^20^, thereby generating the optimized model of structures (Fig. 2).

### Molecular docking simulation

Molecular docking simulation by Autodock (ver. 4.2)^21^ was used to investigate for virtual screening of riluzole and edaravone and inspection of interaction with PFN-1 (at 200 ns) of molecular dynamic trajectories.

This method is partly used to estimate the activity of various molecules that accelerate the process before drug synthesis and during drug discovery. This technique is based on the atomic structure and the interaction of atoms relative to each other. In this study, molecular structures were set for 250 × docking runs with an appropriately necessary algorithm to analyze the displacement at the PFN-1 surface. During these steps, the molecular structures were flexible and found their ideal sites for binding at the PFN-1 molecule. Molecular mechanics are set to suit and calculate the ligand binding energy when interacting with macromolecules. We used the Genetic algorithm-based method for docking, with the program running on a Linux operating system supercomputer system with a total of 16 cores, 32 logical cores of CPU, and 1 compatible GPU. Ubuntu distribution (Linux operating system), (ver. 16). By the Autogrid4 section of Autodock4 Suite, (ver. 4.2), electrostatic interactions and pre-calculations of grid maps, were obtained for each atom. To adjust the size of the box to cover the PFN-1, a grid map consisted of 110 × 100 × 80 Å points around the PFN-1^WT^ before 200 ns and 120 × 100 × 80 around PFN-1^WT^ after MD simulation, and Å grid spacing of 0.375 Å (fourth of the carbon–carbon bond length) was used. The center of the grid was set to the coordinates of the PFN-1 and for each mutation, the respective box sizes were determined.

For flexible PFN-1-ligand docking, the Lamarckian Genetic Algorithm was examined for finding the optimized condition for docking structural outcomes. The results were evaluated and analyzed by UCSF Chimera, Autodock suite, and Ligplot software program which automatically generates schematic 2-D representations of protein–ligand complexes from standard protein data-bank file input used to generate schematic diagrams^22^.

### Mutant PFN-1 proteins

To find the mechanism of PFN-1 toxicity and to explain the harmful and deleterious effects of the point mutation-induced changes to the PFN-1 protein^23^, we created four of the mutant structures reported on the PFN-1 in ALS^5^, primarily to investigate the structural changes of the protein and then examine the functional changes of protein due to mutations using UCSF-chimera command line to create point mutation. The four mutations studied here are located near the actin-binding domain of the human PFN-1. Point mutation modifications were implemented by the Swapaa command code (one or more protein residues to change can be specified in a single command) in the UCSF Chimera command line, ver. 1.13 (Supplementary Table S2).

The main theme for this study was to focus on the structure of PFN-1 including all point mutations in the actin-binding area. The post-mutational structure evaluation involves only a limited number of laboratory experiments^2,24^. Notably, the validation of the point mutation on the protein crystal structure located in the PFN-1 is confirmed after molecular dynamics simulation. In the motion reconstruction and protein residual dynamics, an equilibrium for mutant residue needed to be established in terms of energy and spatial geometry with other surroundings. It may be possible that mutation in an amino acid alters the secondary structure of the protein or alters the active site or regulatory position of the protein^25^. MD simulations at 25, 50, and 100 million steps, which takes 50, 100, and 200 ns, respectively, according to the method described in the previous section, was done by the Gromacs program which is a molecular dynamics package mainly designed for simulations of proteins, lipids, and nucleic acids (2018) for four mutated structures similar to the PFN-1^WT^ structure. In the first step, a detailed structural examination of the mutant PFN-1 was performed by visual analysis with the help of structural display tools. The next step was to find changes in the PFN-1 function due to each mutation, using molecular docking simulation methods. To find these changes, we collected data with 250 × docking runs for PFN-1 binding to each structure.

## Results and discussion

### Mutation impacts PFN-1 protein structure

ALS is a complex degenerative disorder with genetic and environmental components, and it has been difficult to conceptualize and treat it. To evaluate the impact of each mutation, we analyzed the modified structural characteristics of PFN-1. We have represented these by Root-Mean Square Deviation (RMSD)^26,27^, backbone (basic structure), and sidechain (showing amino acid residues), Root Mean Square Fluctuation (RMSF), and evaluated based on the position of alpha carbons (Cα) using 50 ns and 200 ns MD simulations^28^. These are indicated as backbone data, the radius of gyration (Rg) and were analyzed throughout the trajectory and followed up with visual analysis. To further demonstrate and better show the exact timing of the deviation in the molecule, we have produced short movies (see Supplementary Section) on the structural changes where the largest deviations occur to help in the visualization of the graphical data presented in the figures and diagrams.

### PFN1 point mutations on the actin-binding domain

The loss-of-function in PFN1 due to Cys 70 to Gly point mutation leads to the toxicity that kills motor neurons in ALS. Cys is a polar amino acid with uncharged radical groups (R can be any group in which a carbon or hydrogen atom is attached to the rest of the amino acid residue) changed to a nonpolar amino acid with the smallest R group. The 3D structure of PFN-1 shows that the location of Cys 70 is on the beta-sheet structure and changing it to Gly on this residue may not destabilize the beta-sheet but it will cause a loss or inability to form any disulfide bond, which would cause a detrimental effect on the proper folding and interaction of other residues needed for the quaternary structure and linking to other proteins such as actin.

The Met 113 to Thr mutation is also toxic in ALS, and it is a nonpolar amino acid with an aliphatic R group changed to a polar amino acid with an uncharged R group. This mutation is also located on the beta-strand structure. The presence of amino acid residues with a hydroxyl group increases the hydrogen bonding capacity.

The Glu 116 to Gly is another ALS linked mutation, which is a negatively charged R group changed to a nonpolar amino acid with the smallest R group. This change would influence the interaction of the neighboring residues and impact the three dimensional (3-D) structural integrity.

**Figure 3:**
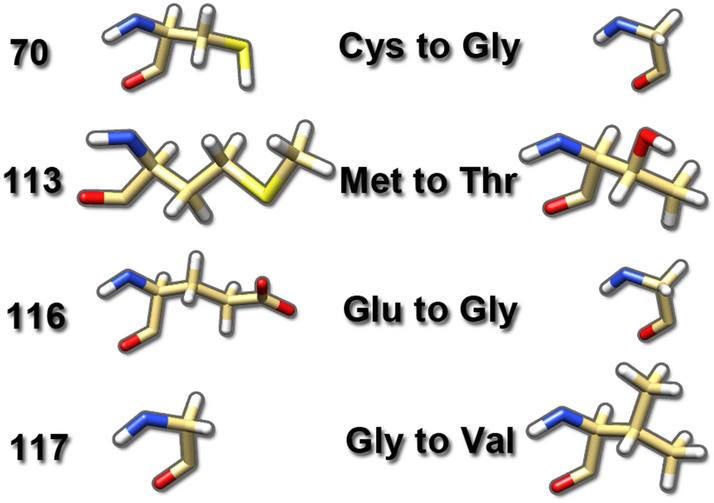
The four amino acid residues are located in the actin-binding domain of PFN-1. The Cys 70 to Gly is a polar amino acid with uncharged R groups changed to a nonpolar amino acid with the smallest R group. The Met 113 to Thr mutation is a nonpolar amino acid with an aliphatic R group changed to a polar amino acid with an uncharged R group. The Glu 116 to Gly is a negatively charged R group changed to a nonpolar amino acid with the smallest R group. The Gly 117 to Val is a nonpolar aliphatic residue that is substituted to Val, with the same nonpolar aliphatic R group.

The Gly 117 to Val is considered toxic in ALS as it is proven in in vitro and in vivo models. Gly is a nonpolar aliphatic residue that is substituted to Val, and although the Val has the same nonpolar aliphatic R group, this point mutation reduces the conformational flexibility of the loop region between the alpha-helix and beta-strand (Fig. 3).

**Figure 4:**
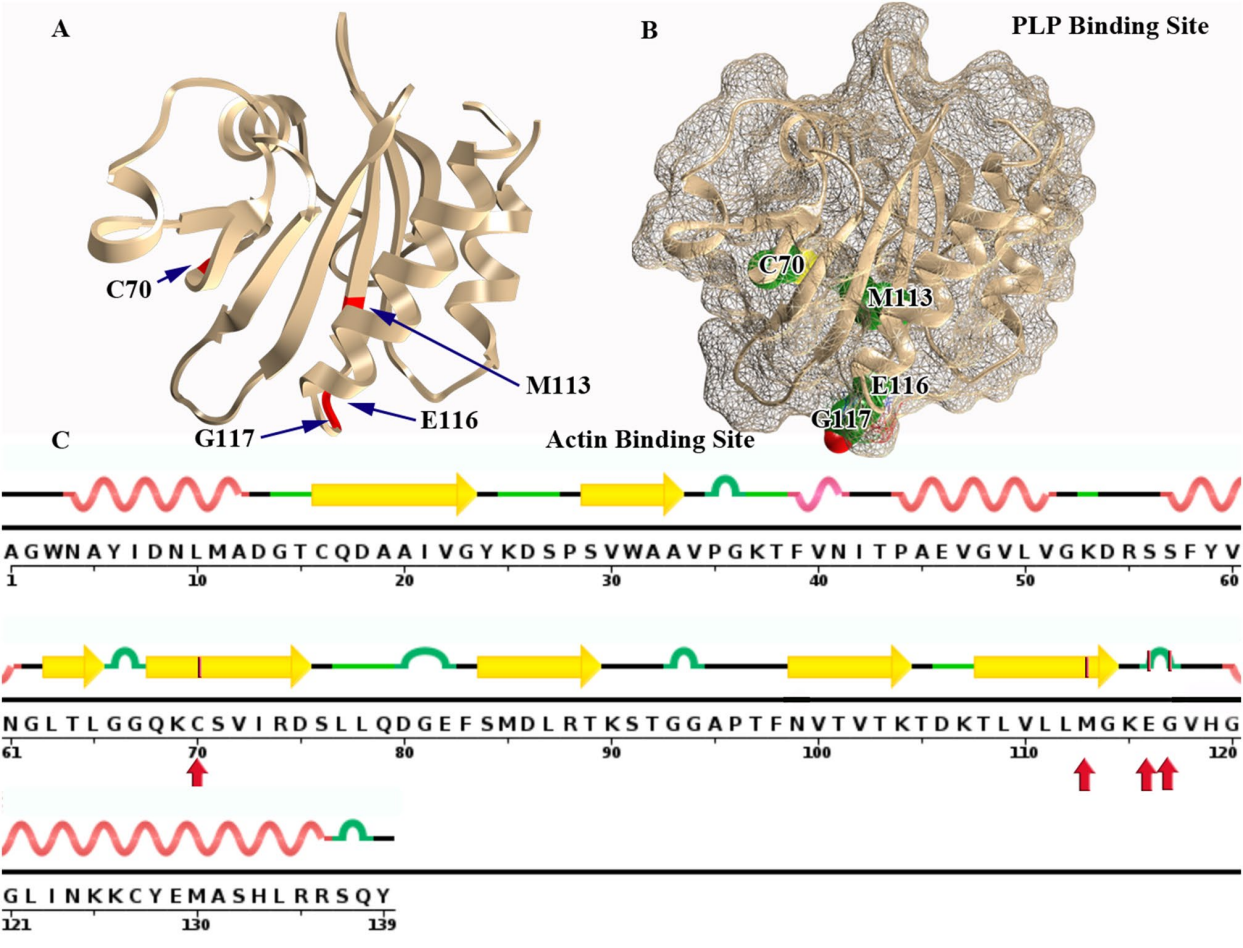
The position of the mutated 4 amino acids in PFN-1 associated with pathogenic injuries in ALS. (**A**) Ribbon view showing the location of the reported mutated 4 amino acids. (**B**) Surface views of the structure and sphere views of the actin-binding domain showing the amino acids E116, G117 located on the surface. Amino acids C70 and M113 are located deep inside the structure. (**C**) Secondary structure of the protein sequence and the position of the mutated amino acids in the polypeptide chain. The C70 and M113 are located on the beta-strand domain and E116 and G117 are on the turn.

The location of each mutation in a fully folded PFN-1 structure (some on the surface and some deep in the structure) with annotation of the properties of each amino acid residue is listed in Fig. 4.

### RMSD measures for the comparison of two molecular ensembles

**Figure 5:**
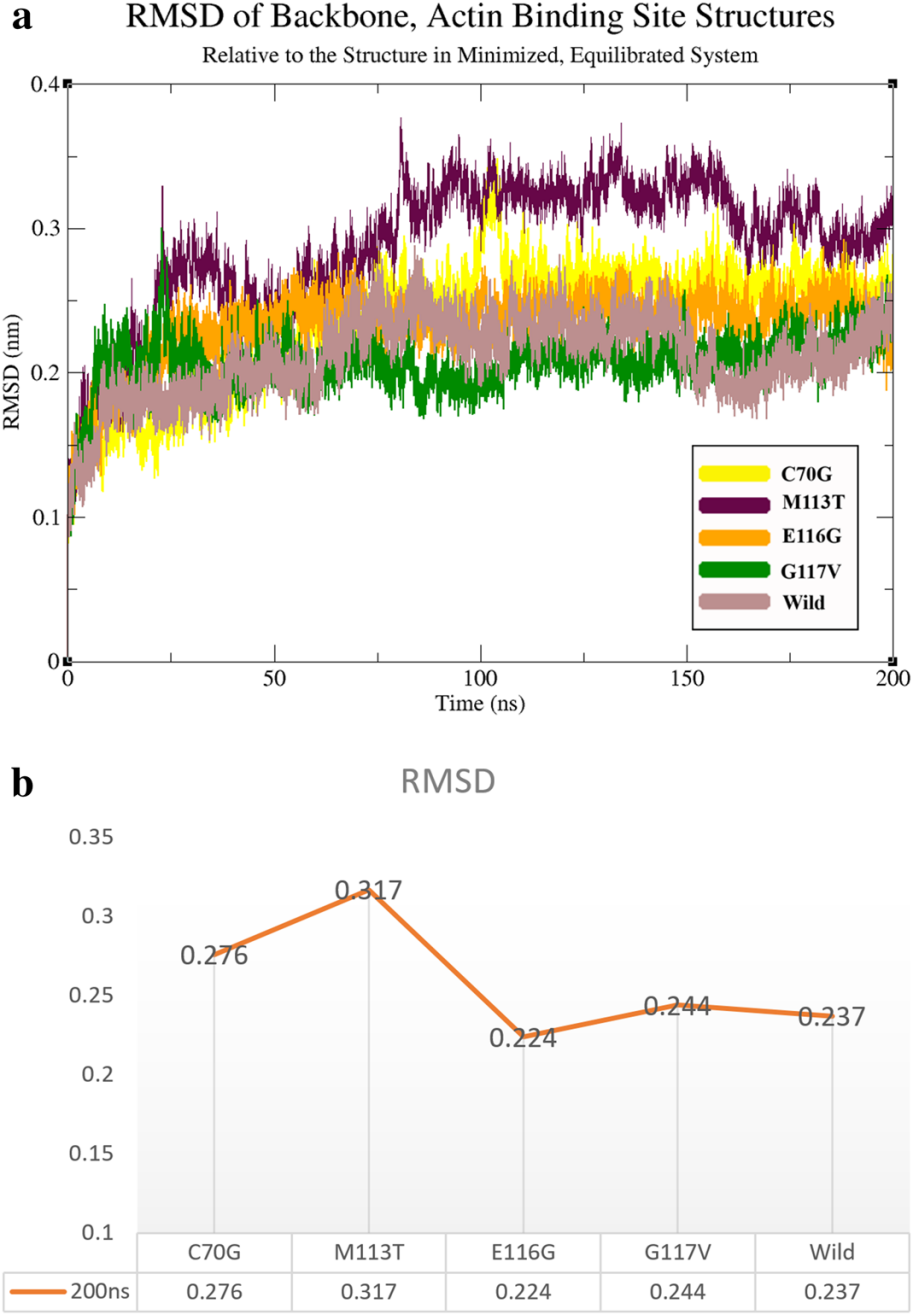
The RMSD data generated from a triplicate runs for 200 ns. (**A**) RMSD of structures after 200 ns relative to the structure in a decent energy-minimized, equilibrated system. (**B**) Compared to RMSD values for 200 ns.

The RMSD diagrams represent the spatial displacement rate and moving parts of the protein model during the simulation, and it serves as a measure of protein stability due to a mutation. The smaller the value of RMSD during the simulation translates to the lower protein stability. During each simulation period, compared to the initial (crystal) state of the simulation, and only with respect to the major atoms of the molecule, amino acid units were calculated for each structure with and without a mutation^29^. The RMSD data generated from a triplicate runs for 200 ns each and compared to the state of the changes with the original structure of the crystal as shown in (Fig. 5). In a short movie, we show the timing and area of the molecules with the largest deviations, wherein C70G movie at 90–110 ns, M113T at 5–25 ns, E116G at 0–20 ns, and for G117V at 10–25 ns are in the display (Supplementary Movie).

### Radius of gyration (Rg) calculations and interatomic distances during MD simulations

**Figure 6:**
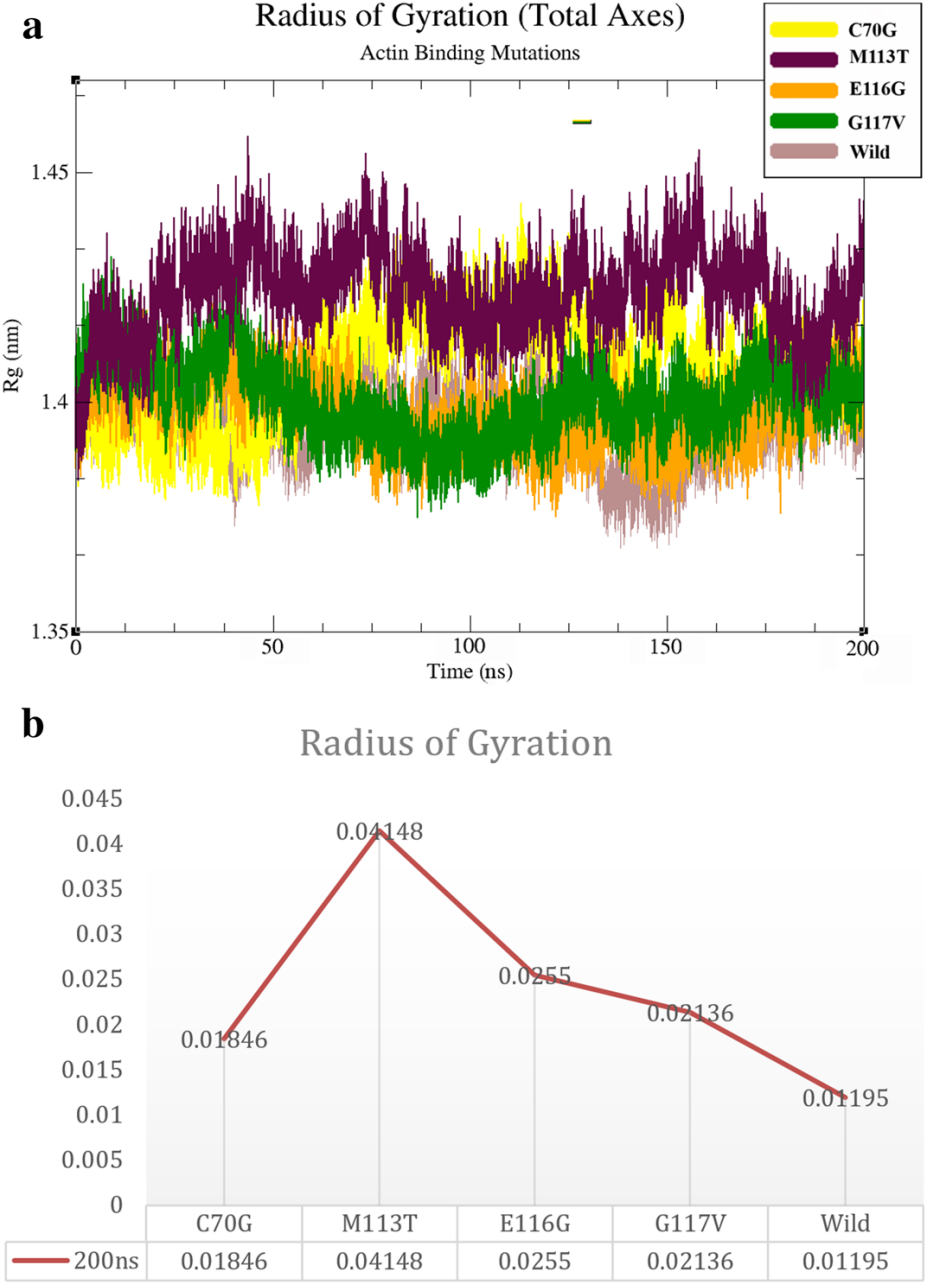
The Radius of gyration for wild-type and all mutant structures After 200 ns MD simulation. (**A**) Changes in the radius of gyration of the wild-type structure and all mutant structures in an outline, each marked with different colors. (**B**) Rate of variation of Radius of gyration.

To find deviation in the parameters relating to the structure of proteins in terms of compression and density as a measure of the stability of the protein after the MD simulation were analyzed. The Rg data globular proteins are based on the principle that the more compact the protein is, the more stable it will be^30^ making Rg is suitable to investigate the characteristics for simulating protein structure that we ran for 50–200 ns MD simulation and measured the radius of gyration. The rate of change for M113T is 0.04 nm and the rate of change of RG for C70G is 0.01 nm. The E116G and G117G have Rg changes close to 0.02 nm (Fig. 6).

### Root mean square fluctuation (RMSF)

**Figure 7:**
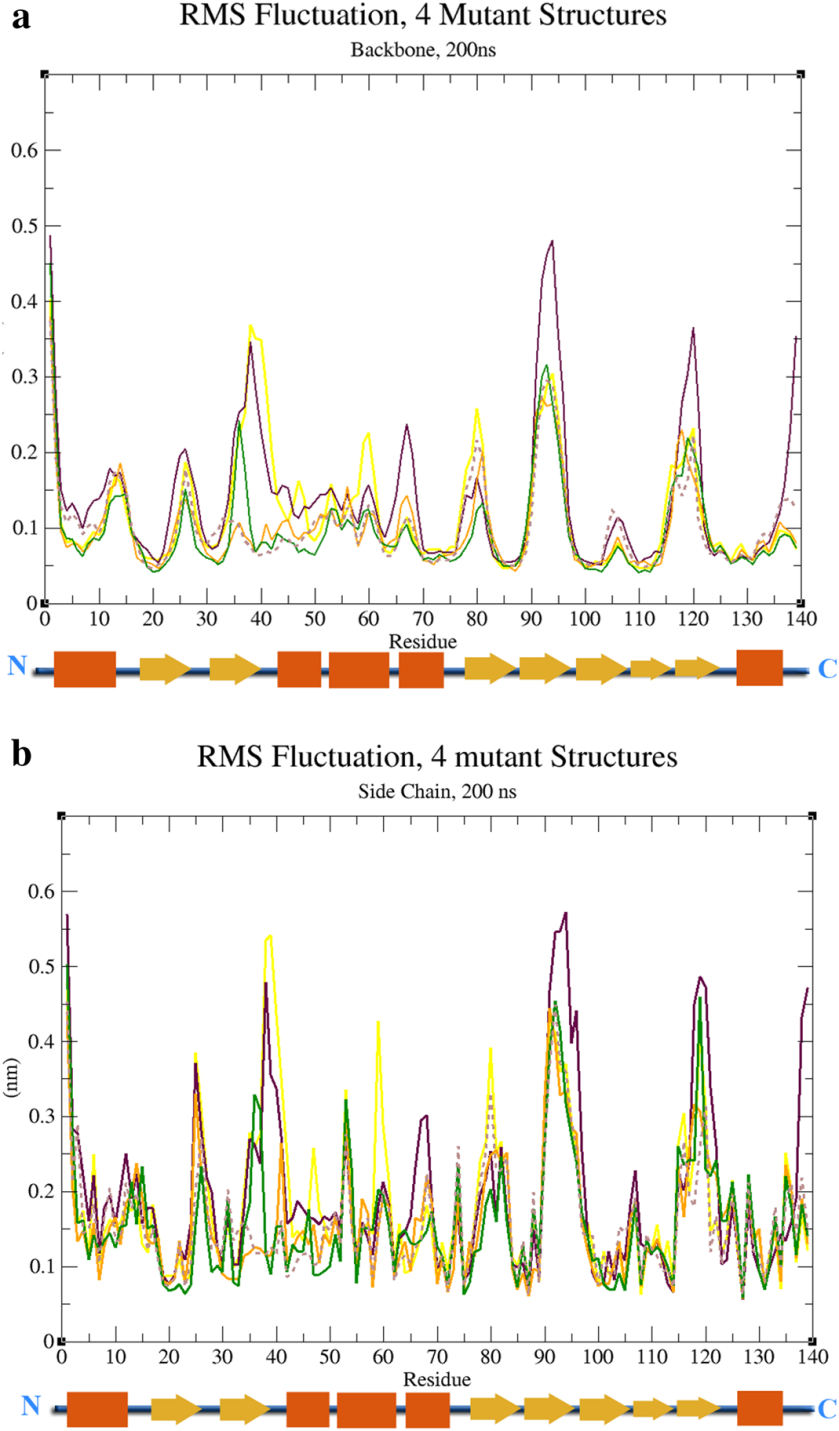
Root Mean Square Fluctuations (RMSF) of backbone and side-chain atoms versus residue number of the WT, and all mutant structures. (**A**) RMSF structures based on the backbone fluctuations of all structures. (**B**) RMSF structures are based on side-chain fluctuations of all structures.

Investigation of the dynamic behavior of Cα atoms in the structure of proteins contains sufficient information to investigate the impact of each motion that reflects in the general motions of the structure. To find the changes in the motion and structural fluctuation we used Cα oscillations (backbone) in an RMSF test. We found the rate of change in fluctuation for M113T to be 0.5 nm maximum in the backbone, 0.6 nm in sidechain in the area of 90–100 amino acid sequences. The rate of change fluctuation for C70G was 0.35 nm in the backbone and 0.55 nm in sidechain in the area of 35–45 residues. The rate of change in the fluctuation for E116G is 0.35 in the backbone and 0.45 nm maximum in the sidechain and the area of 90–100 residues. In G117G we found the rate change of fluctuation to be 0.35 nm in the backbone and 0.45 nm in the side chain with two peaks in the area of 90–100 and 115–125 residues (Fig. 7).

### Superimposition of PFN-1 structure

In order to compare the PFN-1 structure affected by mutation after MD simulation to the PFN-1^WT^, it was needed to set up, train, and observe their differences by overlapping the initial structure of the crystal over the final structure obtained after the simulation. We generated data and analyzed it with the goal in mind to determine and measure the structural changes in the protein structure using 200 ns simulations. Data show an overlap in the state of the primary crystal structure and each of the mutated structures.

**Figure 8:**
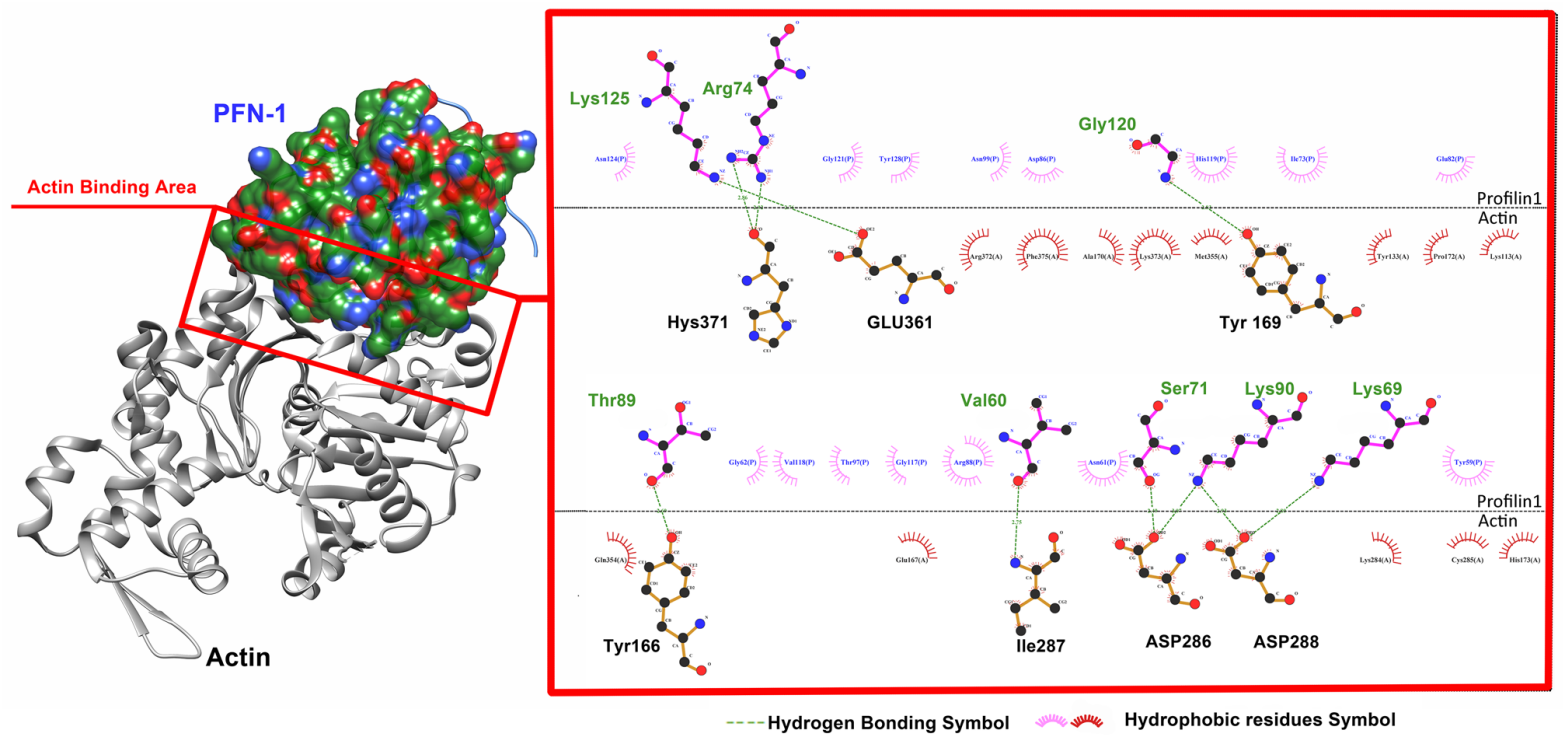
Identified hydrogen bonding and hydrophobic interacting amino acid residues in the actin-binding domain. The amino acid residues Arg 74, Gly 120, Lys 125 and Thr 89, Val 60, Ser 71, Lys 90, and Lys 69 were found to participate in hydrogen bonding ( ). On the actin side, residues are His 371, Glu 361, Tyr 169, Tyr 166, Ile 287, Asp 286, and 288. The amino acid residues involved in the hydrophobic interaction ( ) are identified and noted in the figure.

Visual analysis, before and after MD simulations, allowed us to test our hypotheses, examine protein function and enabled us to corroborate our data with previously published laboratory and clinical data. In the visual analysis of PFN-1 in its crystallized form, the protein binding segments to other structures were precisely pinpointed and the amino acids that play a role in binding to actin are identified^31^. We have identified Hydrogen bonding and hydrophobic interaction amino acid residues in the actin-binding domain. The amino acid residues, Arg 74, Gly 120, Lys 125 and Thr 89, Val 60, Ser 71, Lys 90, and Lys 69, are found to participate in hydrogen bonding. The counter binding residues that form actin molecules are His 371, Glu 361, Tyr 169, Tyr 166, Ile 287, Asp 286, and 288. The amino acid residues participating in hydrophobic interaction are identified and shown in (Fig. 8)^32^.

**Figure 9:**
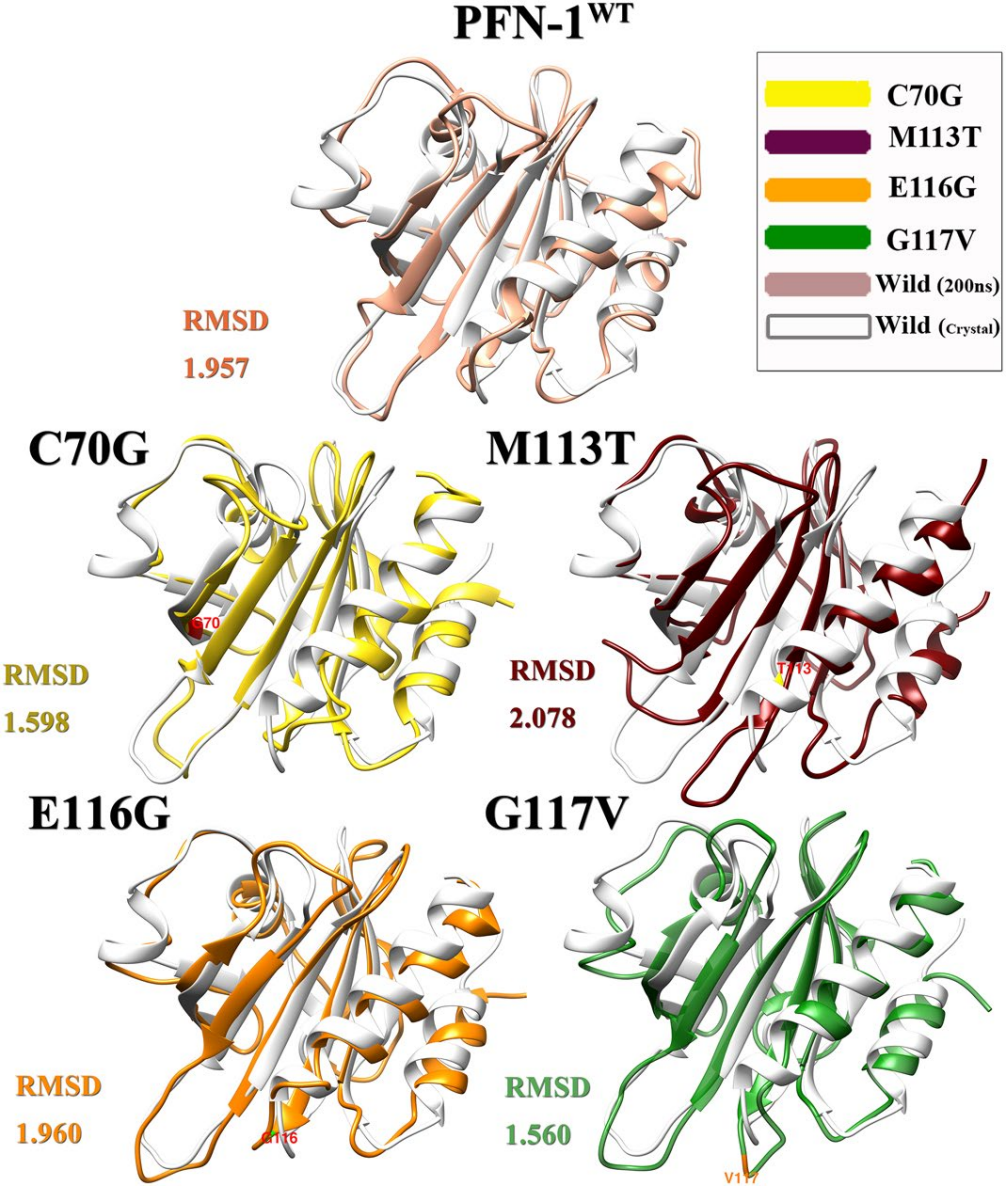
Superimposition of PFN-1^WT^ (white) over the mutant structures (the predefined colors) after 200 ns MD simulation. In this case with RMSD 1.957 which is related to the wild structure of PFN-1 before and after 200 ns MD simulation, The RMSD of M113T mutations with a value of 2.078 is larger than the PFN-1^WT^ structure and the C70G and G117V mutations with a value of 1.560 and 1.598 respectively, are less than the PFN-1^WT^, while RMSD value for E116G is almost equal to the PFN-1^WT^.

**Figure 10:**
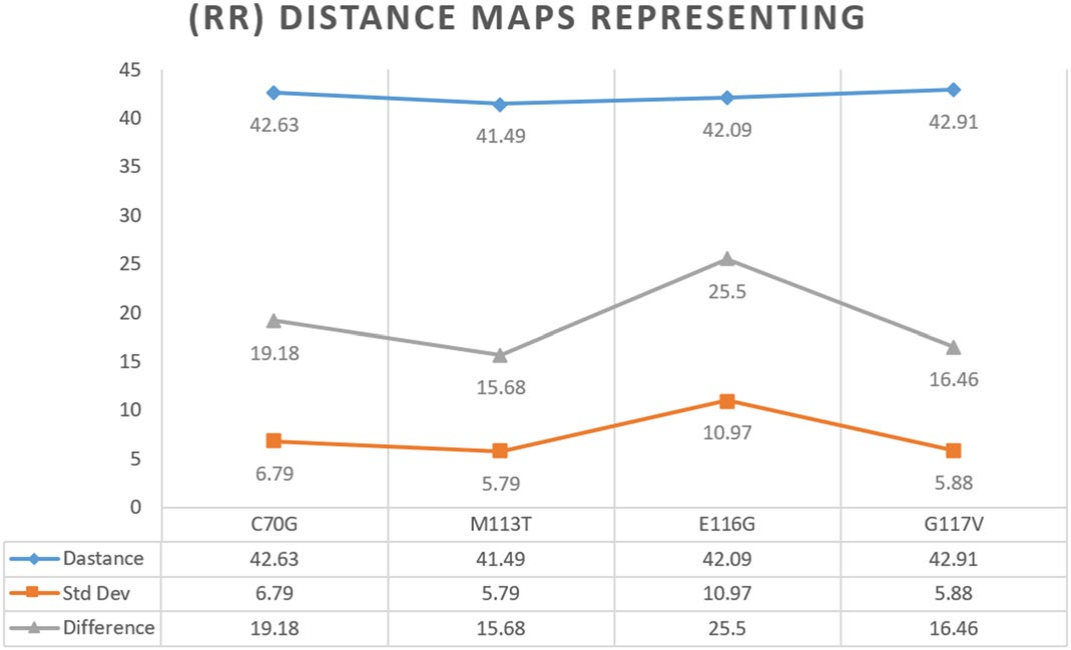
Difference RRDistMaps (for viewing and comparing protein distance) representing the difference between the PFN-1^WT^ and mutant forms of PFN-1. The difference between the Wild and mutant structures at the end of 50 ns MD is 20.27 for the E116G mutation, which is 11 for the C70G mutation, 13.4, and 15.21 for the G117V and M113T mutations, respectively.

The wild-type structures are coded white in Fig. 9 and refer to the crystal structure, and the predefined colors for the mutant structures as given in the figure legends (Fig. 9). This figure is created using data from RMSD, residue-residue distance maps (RRDisMap). The RMSD values (in nm) for C70G, M113T, E116G and G117V are 0.159, 0.237, 0.272, and 0.231, respectively. Structural superimposition and difference in RRDisMap were calculated for selected conformers by their evaluation of the RRDistMap. The α-helices, β-strands, and turns, whose interface form the core of the domain are expected to differ for the wild-type and mutant PFN-1 structures. The differences in wild-type and mutant PFN-1’s core structure was further evaluated. This was done by calculating the overlapped area of residues in the secondary structures, such as helices. The UCSF chimera program was used and the calculated parameters of distance, and the standard deviation denoted in the analysis as shown in (Fig. 10)^33^.

Superimposition of wild-type and mutant 3-D structures was created based on 1FIK with original crystal structure from protein data-bank. Those data points were used as a base and the sequence data entered to generate 3-D structures for the mutants with 200 ns MD simulations. The changes in the RMSD values related to the wild-type structure of PFN-1 before and after the simulation were determined. The RMSD value for wild-type was found to be 1.957 nm and the value for M113T was 2.078 nm which is greater than the wild-type. The C70G and G117V mutations’ RMSD values were 1.560 and 1.598 nm respectively. The RMSD value of E116G is very close to the wild-type value (Fig. 9) and in acceptable cut-off range, which is consistent with the clinical observation that this mutation was reported to be benign, in contrast to other mutants that were found to cause ALS^4^.

### The impact of the mutation on the function of PFN-1

To investigate the effect of the mutation on the PFN-1 function, we used the "molecular docking to ensembles of protein structures approach followed by MD simulations" as described in the “Methods and materials” section. The docking energy of riluzole and edaravone with PFN-1 to determine whether they interact, the degree of interaction, and at which location was first characterized using PFN-1^WT^ and mutant forms^34^.

The docking data for each molecule listed in three rows (Supplementary Table S3). The first row (from left to right) refers to the lowest binding energy state (most stable), shown as a negative value, and the second number refers to the repetitions of distinct clusters at the lowest binding energy. The second row belongs to the docking binding energy with the highest number in the cluster (abundance in cluster) followed by the number in clusters per mutation. The third row refers to the values that are indicating less favorable and unlikely conditions/conformation to occur as the negative binding energies tend to be higher in value. Therefore, the binding energy value and the number in clusters are shown in (Supplementary Table S3).

### Capturing, modeling, and optimization of structures

**Figure 11:**
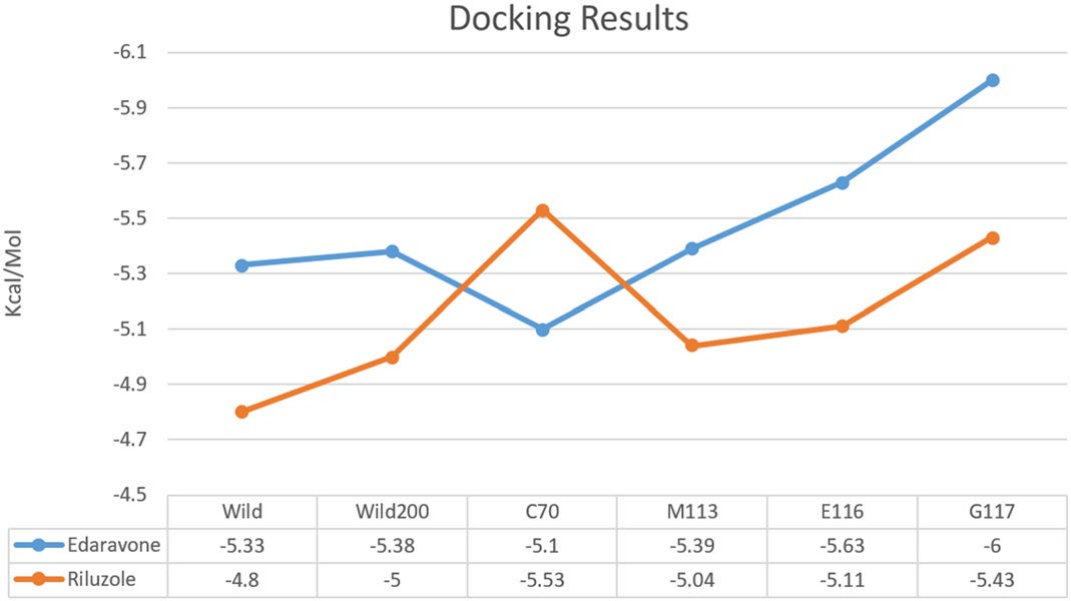
Binding energies (Kcal/Mol) of mutant forms of PFN-1 and PFN1^WT^ vs the two FDA-approved drugs for ALS.

**Figure 12:**
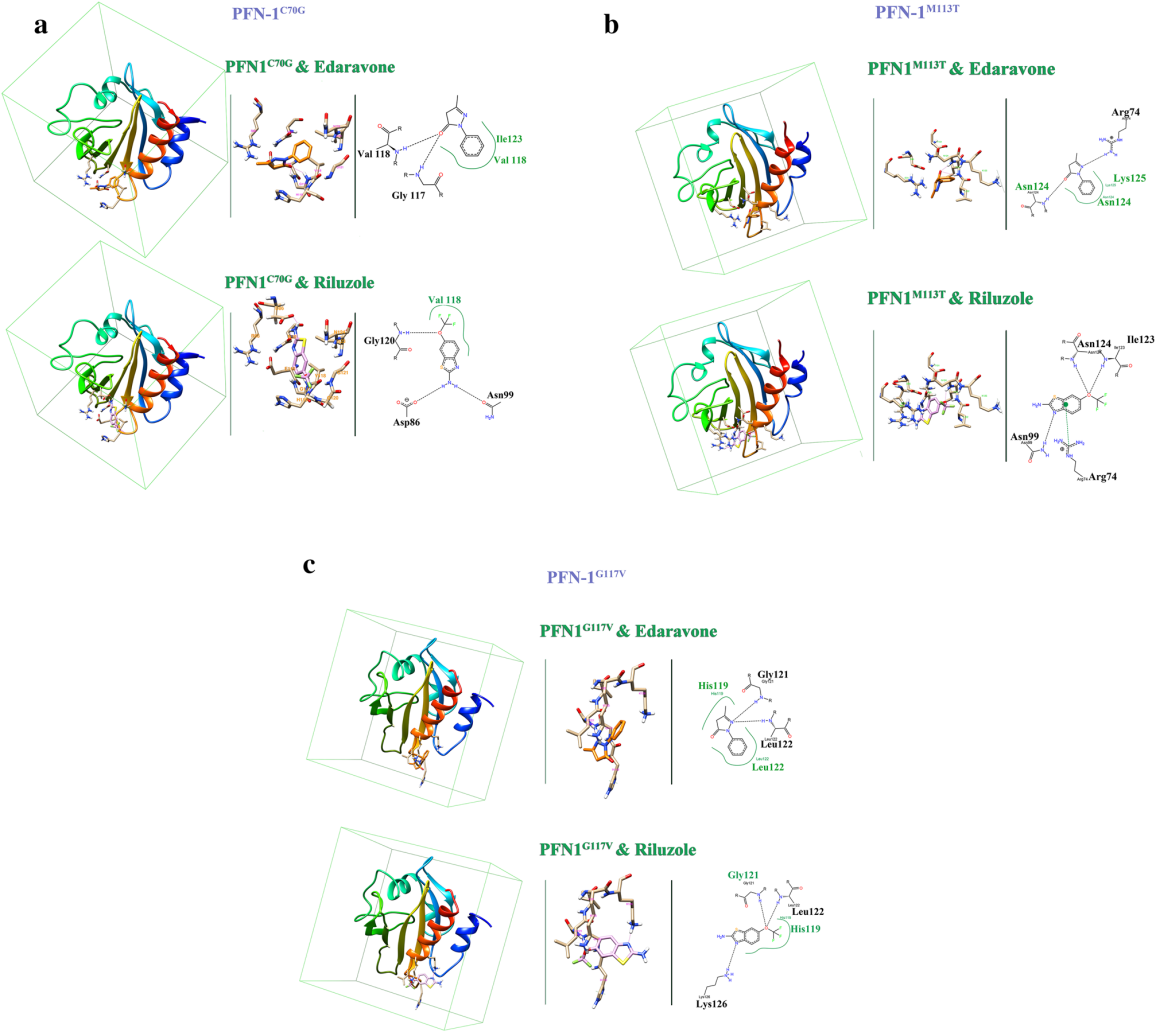
Possible Interaction of FDA-Approved Drugs for ALS; Edaravone, Riluzole with mutant PFN-1 at C70G, M113, and G117 residues determined with molecular docking simulation are shown in ribbon, sticks, and molecular view. (**a**) Riluzole and Edaravone interacting with C70G mutant PFN-1 (**b**). Riluzole and Edaravone interacting with M113T mutant PFN-1. (**c**) Riluzole and Edaravone interacting with G117V mutant PFN-1.

We describe our findings in capturing and preparing small molecule structures (riluzole and edaravone) to perform molecular docking simulations based on the previously discussed topics as shown in (Fig. 2). The results of molecular docking simulations of PFN-1^WT^ and mutant PFN-1 structures over the small molecular structures were extracted with details (Supplementary Table S3) and (Fig. 11). We have presented analytical images of the molecular docking simulation (Fig. 12a–c). The steps and parameters of simulating molecular docking are described above.

### Conformational and structural changes

The in silico evidence drives our speculation that structural changes due to a given mutation (e.g. E116G) disturb the protein structure in the actin-binding region. Therefore, the actin-binding region is critically important and highly vulnerable to mis-conformation and explains the loss of binding affinity to actin molecules. The Rg changes indicate the value of protein structure compression (Fig. 6). The RRDistMaps variations indicate the largest difference in the E116G mutation as shown in (Fig. 10). Interestingly the RMSD data consistently show the largest change in E116G mutation.

Furthermore, RMSD data analysis revealed that the M113T mutation is located deep in the central region of PFN-1 compared to the wild-type crystal structure listed in (Supplementary Table S1). This mutation is not juxtaposed to the actin-binding domain; however, it may alter the 3-D structural integrity and stability of the PFN-1.

It is of great interest that RMSF analysis of MD simulations demonstrated altered dynamic properties for these mutant PFN-1 that we studied. The largest alteration in the protein dynamics was found to be caused by G117V mutations.

### Functional changes in PFN-1 and interaction of Riluzole and Edaravone

The effects of point mutations on "protein function" according to parameters set in silico and before and after scenarios, which are as shown in (Fig. 12a–c), unravel atomic details of the alteration that could be the basis for “gain-of-function” or “loss-of-function” of PFN-1 in ALS. We have carefully utilized the computer-generated images and checked the binding energy (Supplementary Table S3).

Since ALS patients with the PFN-1 mutation may receive treatment with the current FDA-approved drugs, we sought to examine the interaction of riluzole and edaravone with mutant PFN-1. Our objective was to examine whether the patients with PFN-1 mutations could potentially be able to expect some clinical effect from the available and FDA approved therapies for ALS. These two drugs appear to have an affinity to bind the mutant PFN-1, which, if confirmed, would be of great value for the impact on patients.

For instance, we observed the highest affinity for the PFN-1C71G structure of FDA-approved drugs under two conditions, before and after the mutations. Interestingly, we found that G117V and C70G mutations have the least affinity for these drugs (Fig. 11). Although it is beyond the scope of this study to investigate these differences in the affinity to interact with riluzole or edaravone, a future study may reveal important knowledge on how each mutant PFN-1 or other ALS-causing mutated proteins (e.g. *SOD1* gene, TDP-43 neurofilament or gene encoding FUS) interact with drugs that patients carrying any of these mutations are receiving (Fig. 11). The images of the mutants and wild-type PFN-1 interacting with these two ALS drugs are fully analyzed and depicted in the Fig. 12a–c. The in silico tools we employed for our investigation in this study were instrumental in the generation of these invaluable data and enabled us to learn how each of the four mutations contributed to the structural deviations. Our data appear to be consistent with the in vitro and in vivo observations reported by us and others. In contrast, others employed algorithmic programs such as PhD-SNP, PMUT, PolyPhen- 2, SIFT, SNAP, SNPS&GO, SAAP, nsSNPAnalyzer, SNPeffect4.0, and I-Mutant2.0 to predict the functional and stability of mutant PFN-1 and their conclusion was to re-confirm what was already known. Therefore, the outcome of these studies was useful although impacted by a shorter simulation of 100 ns as the study by Pereira et al.^35^. The approach that we have used is popular and frequently utilized in protein structural analysis adding to our confidence for the choice of tools that allowed us a study with a greater depth that revealed nuances that weren’t known.

## Conclusion

Biophysical and MD simulation analysis series of wild-type and mutant PFN-1 proteins with the variation revealed critical information that we have generated on the conformational and functional changes in mutant PFN-1. This may elucidate the underlying mechanism of neurotoxicity observed in ALS. Further investigation and confirmation of these in silico data can shed light on the toxicity of mutant PFN-1 and may turn highly informative in pre-clinical studies for the development of a novel therapy for ALS patients with mutant PFN-1, as well as possible treatments to prevent in sALS cases. Once confirmed, this approach may be used on other ALS-causing mutants and suspected proteins to be involved in the neurotoxicity of both sALS and fALS. The in silico method has great power and real potential to facilitate the investigation of the changes in the mutant protein outside the wet laboratory^7^. It also enables researchers to develop and test variety of hypotheses using computer programs otherwise wouldn’t be possible in the wet laboratory.

This study on the four ALS-causing point mutations in the actin-binding domain of PFN-1 protein reveals the structural deviations in the structure and the possible adverse impact on the PFN-1 interaction and its function. Altogether, our analysis using modeling and simulation tools detected important changes in the PFN-1 which revealed atomic-level mechanisms of PFN-1 protein structure. The structural deviations in the overall structure measured by RMSD, RMSF, the Radius of Gyration, RRDisMap, as a new series of parametric indexes into the stability and 3-D formation are interesting and reveal nuances for the mutant PFN-1. This data also shed light on the quaternary structure of the protein that may impact the function(s) of PFN-1 and could partly explain the toxic gain/loss-of-function as the mechanisms in the neurodegeneration in ALS. Since there are two FDA-approved drugs for ALS, we investigated whether these drugs would interact with mutant PFN-1. We found both new therapeutic medications, riluzole, and edaravone interact with mutant PFN-1, where restoring the stability of the PFN-1 structure due to the interaction of these drugs may take place. Therefore, ALS patients carrying PFN-1 mutations may inquire about getting these drugs prescribed which can attenuate disease progression.

## Supplementary Information

Supplementary Information.

Supplementary Video 1.
