# Supplementary material for: In silico studies reveal structural deviations of mutant profilin-1 and interaction with riluzole and edaravone in amyotrophic lateral sclerosis
Source: Sci Rep. 2021 Mar 25;11:6849. doi: 10.1038/s41598-021-86211-4 (PMC7994392; doi:10.1038/s41598-021-86211-4)
Supplement: Supplementary file 1 — Supplementary Information. [file 41598_2021_86211_MOESM1_ESM.docx]

**Supplementary Material Section**

In Silico Studies Reveal Structural Deviations of Mutant Profilin-1 and Interaction with Riluzole and Edaravone in Amyotrophic Lateral Sclerosis

Ahmad Shahir Sadr^1,3^, Changiz Eslahchi^1,2§*^ Alireza Ghassempour^3^, Mahmoud Kiaei^4,5§*^

Table S 1. List of PFN-1 crystal structure codes in the protein data-bank.

|  | Entity ID | C ID | C Length | R Date | R (Å) | MW |
| --- | --- | --- | --- | --- | --- | --- |
| 1 | 6NBE_3 | P | 140 | 4/15/2020 | 2 | 85.09 |
| 2 | 6NBW_3 | P | 140 | 1/29/2020 | 2.5 | 84.8 |
| 3 | 6NAS_3 | P | 140 | 1/29/2020 | 2.9 | 85.14 |
| 4 | 4X25_1 | A, B | 140 | 6/10/2015 | 2.23 | 15.04 |
| 5 | 4X1M_1 | A | 140 | 6/10/2015 | 2.17 | 14.99 |
| 6 | 4X1L_1 | A | 140 | 6/10/2015 | 2.16 | 15.17 |
| 7 | 3CHW_2 | P | 139 | 8/19/2008 | 2.3 | 58.5 |
| 8* | 2PBD_2 | P | 139 | 11/13/2007 | 1.501 | 61.47 |
| 9 | 2PAV_2 | P | 139 | 10/23/2007 | 1.8 | 58.72 |
| 10 | 1CJF_1 | A, B | 139 | 7/7/1999 | 2.3 | 33.27 |
| 11 | 1CF0_1 | A, B | 138 | 7/6/1999 | 2.2 | 30.82 |
| 12 | 1AWI_1 | A, B | 138 | 10/28/1998 | 2.2 | 30.73 |
| 13 | 1FIL_1 | A | 139 | 11/8/1996 | 2 | 15.04 |
| 14* | 1FIK_1 | A | 139 | 11/8/1996 | 2.3 | 15.03 |
| 15 | 1PFL_1 | A | 139 | 3/31/1995 | NMR | 14.94 |

*. Indicate the protein codes analyzed in detail in this study.

Table S 2. Swapaa command code in the UCSF Chimera

| **Swapaa Gly #0:70.a** | **Swapaa Thr #0:113.a** |
| --- | --- |
| **Swapaa Gly #0:116.a** | **Swapaa Val #0:117.a** |

Table S 3. Docking scoring energies of Riluzole and Edaravone with PFN-1 variant.

| PFN-1 | Riluzole | Edaravone | Annotation Symbols |
| --- | --- | --- | --- |
| C70G | **-6.16^*^/22 ^Ψ^** | **-6.19/81** | *** Lowest Binding Energy ^Ψ^ Abundant in cluster** |
|  | **-5.54^§^/98^Ψ^/14^€^** | **-6.19/81/12** | **^§^ highest abundant in cluster**  ^€^ **Number of clusters** |
|  | **-3.83^¥^/1^Ψ^** | **-4.37/1** | **^¥^ Highest Binding Energy** |
| E116G | **-5.31/38** | **-5.66/61** |  |
|  | **-4.68/50/19** | **-4.96/74/12** |  |
|  | **-3.68/2** | **-4.60/7** |  |
| G117V | **-4.74/98** | **-4.74/119** |  |
|  | **-4.74/98/16** | **-4.74/119/17** |  |
|  | **-3.78/3** | **-4.09/1** |  |
| M113T | **-5.70/87** | **-5.53/65** |  |
|  | **-5.70/87/21** | **-5.47/77/15** |  |
|  | **-3.92/1** | **-4.15/1** |  |
| PFN1 ^WT^ | -4.76/35 | -5.73/196 |  |
|  | -4.23/43/20 | -5.73/196/8 |  |
|  | -3.87/1 | -4.62/1 |  |
| PFN1^WT^  *After*  *200ns* | -5.91/143 | -5.70/39 |  |
|  | -5.91/143/9 | -5.47/80/9 |  |
|  | -3.85/1 | -4.66/1 |  |
